# Citicoline Eye Drops Protect Trabecular Meshwork Cells from Oxidative Stress Injury in a 3D In Vitro Glaucoma Model

**DOI:** 10.3390/ijms231911375

**Published:** 2022-09-27

**Authors:** Stefania Vernazza, Mario Passalacqua, Sara Tirendi, Barbara Marengo, Cinzia Domenicotti, Diego Sbardella, Francesco Oddone, Anna Maria Bassi

**Affiliations:** 1 Department of Experimental Medicine (DIMES), University of Genoa, 16132 Genoa, Italy; 2 Inter-University Center for the Promotion of the 3Rs Principles in Teaching & Research (Centro 3R), 56122 Pisa, Italy; 3 IRCCS-Fondazione Bietti, 00198 Rome, Italy

**Keywords:** trabecular meshwork, citicoline eye drops, glaucoma, 3D advanced in vitro model

## Abstract

Intraocular pressure (IOP) is considered an important modifiable risk factor for glaucoma, which is known as the second leading cause of blindness worldwide. However, lowering the IOP is not always sufficient to preserve vision due to other non-IOP-dependent mechanisms being involved. To improve outcomes, adjunctive therapies with IOP-independent targets are required. To date, no studies have shown the effect of citicoline on the trabecular meshwork (TM), even though it is known to possess neuroprotective/enhancement properties and multifactorial mechanisms of action. Given that reactive oxygen species seem to be involved in glaucomatous cascade, in this present study, an advanced millifluidic in vitro model was used to evaluate if citicoline could exert a valid TM protection against oxidative stress. To this end, the cellular behavior, in terms of viability, apoptosis, mitochondrial state, senescence and pro-inflammatory cytokines, on 3D human TM cells, treated either with H_2_O_2_ alone or cotreated with citicoline, was analyzed. Our preliminary in vitro results suggest a counteracting effect of citicoline eye drops against oxidative stress on TM cells, though further studies are necessary to explore citicoline’s potential as a TM-target therapy.

## 1. Introduction

Citicoline is the international non-proprietary name for CDP-choline, a nootropic and psycho-stimulant compound. Once absorbed, citicoline is hydrolyzed into choline and cytidine 5′-diphosphate (CDP), i.e., two crucial precursors involved in many physiological pathways. In fact, while choline alone plays a central role in neurotransmitter synthesis (acetylcholine), and in homocysteine reduction, CDP is involved in cardiolipin synthesis. Moreover, both are involved in the Kennedy pathway, which serves as de novo synthesis of phosphatidylcholine (PtdCho), phosphatidylethanolamine, phosphatidylserine, and sphingomyelin [1,2,3]. Therefore, the administration of citicoline, thanks to its multifactorial mechanism of action [1,4,5], has shown its neuroprotective, neurorestorative and neuroregenerative properties in the treatment of several brain disorders, including cognitive impairment and/or behavioral alterations in the elderly, as well as for strokes, traumatic comas, Alzheimer’s Disease (AD), Parkinson’s Disease (PD), traumatic brain injury and glaucoma [6,7,8,9,10].

Citicoline has been used in the treatment of Primary Open Angle Glaucoma (POAG) since the late 1980s, in order to enhance both retinal and visual functions [11,12,13,14,15]. In this regard, citicoline in oral solution was registered in four European countries as a “Food for Special Medical Purposes” with the therapeutic indication “for glaucomatous patients, pharmacologically-stabilized and with a progressive loss of the visual field”. In addition, in the last decade, a topical ophthalmic solution containing citicoline, namely OMK1^®^ (Omikron Italia srl), has also been made available as a coadjuvant to antihypertensive therapy with promising results [16,17].

POAG is a degenerative optic neuropathy that shares its common outcomes with other glaucoma forms, such as the loss of retinal ganglion cells (RGCs) and moderate-to-severe visual field deficits that can progress to blindness.

Today, high intraocular pressure (IOP) is considered the most important POAG-modifiable risk factor. Nevertheless, in addition to POAG forms characterized by IOP within a normal range (i.e., Normal Tension Glaucoma), in some patients, lowering the IOP by topical hypotensive drugs or surgery is not sufficient to save their eyesight [18]. Therefore, although the mechanical strain induced by an elevated IOP seems to be closely related to RGC damage, the precise causes of RGC death in glaucoma have not yet been fully understood [11,12,13,14,18,19].

Given the complex nature of POAG, it is also necessary to consider IOP-independent therapeutic targets in order to prevent RGC death, including the trabecular meshwork (TM), mitochondria and cell membranes [15]. As known, the TM represents the first barrier to the outflow of aqueous humor (AH) through the conventional pathway of the human eye. In POAG, an increase in outflow resistance is related to a mechanism of TM stiffening driven by senescence and apoptosis of the TM cells, as well as to a remodeling of the extracellular matrix [20]. Although the loss of TM functionality may be caused by a number of factors, many studies have demonstrated that local oxidative stress conditions alter the TM so much so as to produce a vicious circle of damage, ranging from cellular aging and chronic inflammation to apoptosis and cell death [21,22,23,24,25]. All these outcomes prove that TM dysfunction is not only responsible for IOP elevation but, in being involved in the secretion of pro-inflammatory cytokines, it may also affect the health of RGCs. In fact, it has been reported that innate immunity is implicated in POAG pathogenesis, including TM fibrosis and RGC death. Moreover, Singh et al. [26] have demonstrated the active role of TM-derived inflammatory mediators in response to the acute Zika virus infection in triggering the early apoptosis of RGCs [27,28]. Therefore, the use of TM-targeted therapies could provide adjuvants to the conventional ones, improving the treatment of glaucoma in those patients in which IOP management alone is not sufficient.

Currently, research in the field of glaucoma mainly uses animal models and bidimensional (2D) culture systems for studying its molecular basis and/or for drug screening. However, both approaches present limits in terms of a lack of species specificity in one case and of tissue complexity in the other. Therefore, the possibility of achieving a greater level of glaucoma knowledge using sophisticated in vitro human-based 3D approaches (e.g., scaffolds, hydrogel-pressurized chambers, organ-on-a-chip technologies, millifluidic devices, and so on) could close the gap between animal models and 2D culture systems [29,30,31,32,33,34].

Our research team carried out a series of studies to set up an in vitro 3D-innovative human-based model combined with millifluidic technologies (IVOM). This model was seen to be not only useful in verifying the effects of oxidative stress on human TM cell (HTMC) behavior [25], but also in evaluating whether HTMCs, subjected to prolonged oxidative stress and pressure elevation conditions, could affect the healthy state of neuron-like cells [35]. The results of our research clearly suggested that oxidative stress had a pivotal role in promoting TM damage, and, above all, that the addition of pressure elevation to the oxidative stress further exacerbated the cellular damage. Moreover, the progressive impairment found in the neuron-like cells (from the SH-SY5Y neuroblastoma cell line) after the prolonged exposure to HTMC-derived experimental culture media suggested that HTMC dysfunction may be responsible for promoting collateral cell damage.

Herein, given that IVOM could be a promising and most useful starting point in addressing pre-clinical investigations, this present study is aimed at highlighting if citicoline eyedrops could be considered also a TM-targeted therapy in addition to the already well-known RGC-targeted one [16,17]. Therefore, starting from the pharmacological function of citicoline, its potential ability to overcome H_2_O_2_-induced damage on HTMCs was investigated in terms of the antioxidant activity and cell viability, the inhibition of apoptosis and pro-inflammatory cytokines, the prevention of acute senescence and the protection of the mitochondrial function.

## 2. Results

### 2.1. Citicoline Counteracts the Cytotoxic H_2_O_2_-Mediated Effect on HTMC

**Figure 1:**
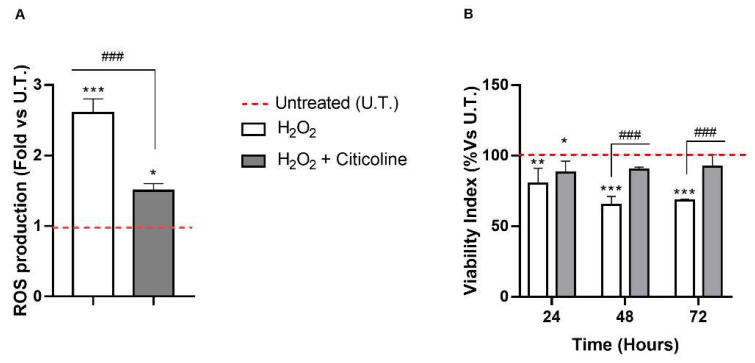
Citicoline counteracts H_2_O_2_-mediated cytotoxicity in HTMC. (**A**) DCF Assay was performed on untreated and treated HTMCs, and fluorescence was recorded at 2 h of experimental procedures. Data are expressed as a fold of ROS production and represent the mean ± SD of three independent experiments, each performed six times. (**B**) The Alamar blue assay was performed on the same sample after 24, 48 and 72 h of daily treatments. Data are expressed as a % of viability and represent the mean ± SD of three independent experiments. *** *p* < 0.001, ** *p* < 0.01, * *p* < 0.05 vs. untreated (UT) HTMCs; ### *p* < 0.001 vs. treated HTMCs (two-way ANOVA followed by Bonferroni post-test).

We previously reported that the damage on HTMCs could have a role in POAG neurodegeneration [35]. Herein, we investigated the effects of citicoline on the ROS production and its protective role in limiting the effect of prolonged exposure to H_2_O_2_ on HTMC viability. As shown in Figure 1A, the early H_2_O_2_-induced production of ROS was significantly reduced by citicoline. Moreover, while the resorufin-reducing capacity of H_2_O_2_-treated HTMCs was significantly reduced starting at 24 h with progressive worsening in the following times (−30%), that of HTMCs treated also with citicoline showed an improvement of the activity of cellular redox enzyme [36] (Figure 1B).

**Figure 2:**
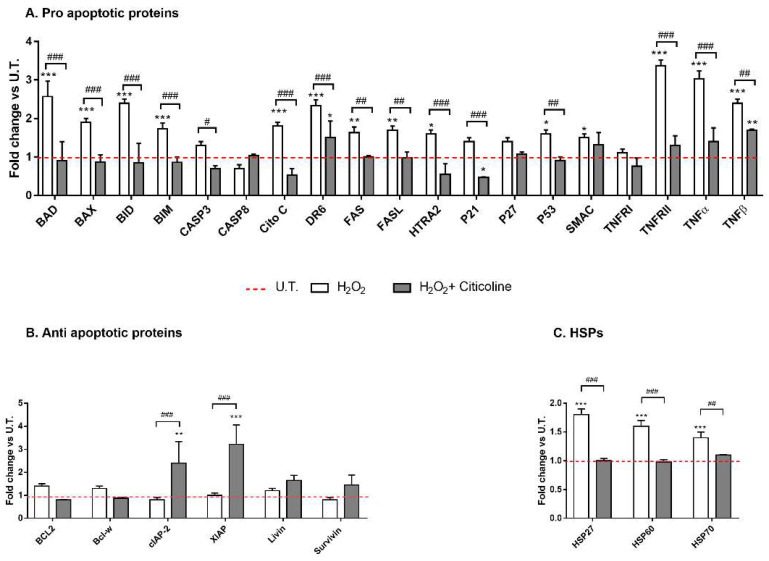
Apoptosis-array analysis. The analyses of (**A**) pro-apoptotic proteins, (**B**) anti-apoptotic proteins and (**C**) HSPs were performed after 48 h of experimental procedure by Human Antibody Array C1 (RayBio C-series). The red dotted line represents the protein level of untreated (UT) HTMCs for each of the proteins examined. Six individual models were arrayed and per experiment the intensity of Positive Control Spot was used to normalize signal responses for comparison results across multiple arrays. *** *p* < 0.001, ** *p* < 0.01; * *p* < 0.05 vs. UT HTMCs; ### *p* < 0.001, ## *p* < 0.01; # *p* < 0.05 vs. treated HTMCs (two-way ANOVA followed by Bonferroni post-test).

Next, two methods were used to evaluate if citicoline was able to inhibit apoptosis. Firstly, antibody array was performed to analyze the changes in intracellular proteins associated with apoptosis. Figure 2A clearly shows that citicoline cotreatment at 48 h significantly reduced the levels of several pro-apoptotic proteins related to intrinsic and extrinsic pathways compared to H_2_O_2_-treated HTMCs. In fact, H_2_O_2_ treatment alone induced an increase in several apoptosis-associated proteins including BAD, BAX, BID, CASP3, CitoC, DR6, FAS, FAS_L_, HTRA2, p53, and SMAC TNFα. Moreover, the levels of two anti-apoptotic proteins involved in the inhibition of death receptor-mediated and mitochondria-mediated apoptosis, namely, cIAP2 and XIAP, were increased only in citicoline-cotreated HTMCs (Figure 2B). However, the levels of Heat Shock Protein (HSP) were increased only in H_2_O_2_-treated HTMCs (Figure 2C). As known, HSPs 70 and 27 upregulation play a cytoprotective role in response to a stressor (e.g., oxidative stress), whilst the HSP 60 induction is related to several pathological human pathways (e.g., mitochondria damage or mitochondria DNA depletion and apoptosis), serving as a danger signal of stressed/damaged cells. The fact that citicoline-cotreated HTMCs exhibited no significant differences with the untreated ones suggests that citicoline protected cells against H_2_O_2_-induced oxidative stress damage.

**Figure 3:**
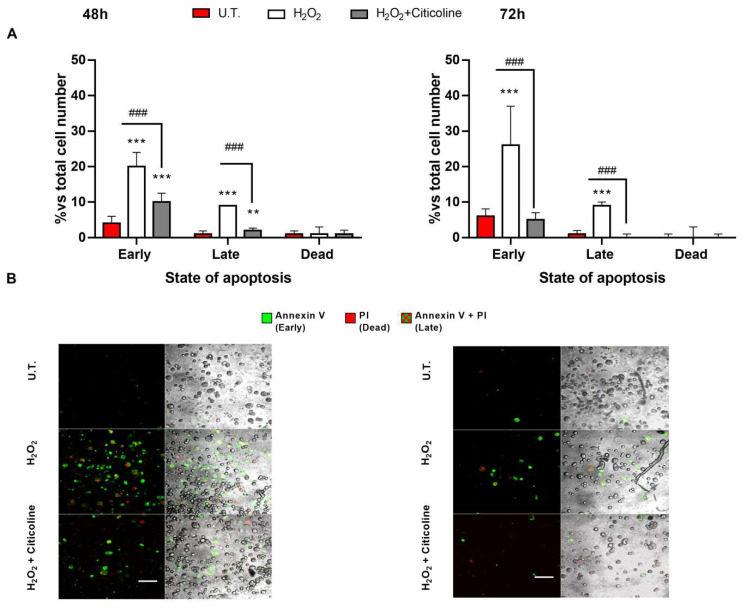
Annexin V binding and PI uptake Annexin V-FITC/PI-staining was used to evaluate the different stages of apoptosis in 3D HTMC. (**A**) The bar chart shows early and late apoptosis and dead cells after 48 and 72 h of experimental procedures. (**B**) Image acquisition was performed using a Leica TSC SP confocal microscope (Leica Microsystem, Wetzlar, Germany). Images depict apoptotic HTMC cells induced by experimental treatments stained with Annexin V-FITC/PI and observed by fluorescence microscopy analysis. The samples were analyzed for green fluorescence (FITC) and red fluorescence (PI). BF images have been added to view the total cell number for each condition (scale bar marker corresponds to 50 µm). *** *p* < 0.001, ** *p* < 0.01 vs. untreated (UT) HTMC; ### *p* < 0.001 vs. treated HTMCs (two-way ANOVA followed by Bonferroni post-test).

The protective effects of citicoline treatment against H_2_O_2_-induced HTMC apoptosis were also investigated by Annexin V binding and PI uptake. In fact, the positivity of the cells to Annexin-V indicates an early apoptosis due to the translocation of PS residues on the plasma membrane (annexin-V-positive cells), while the incorporation of propidium iodide (PI) reflects a cell death. However, the positivity of the cells to both Annexin-V and PI is a sign of late apoptosis. Therefore, a reduction in the rate of both early and late apoptosis was observed at 48 h in citicoline-cotreated HTMCs compared to the H_2_O_2_-treated ones, whereas, at 72 h, citicoline was able to oppose the early apoptosis, thereby preventing the H_2_O_2_-induced cell death of the HTMCs (Figure 3).

**Figure 4:**
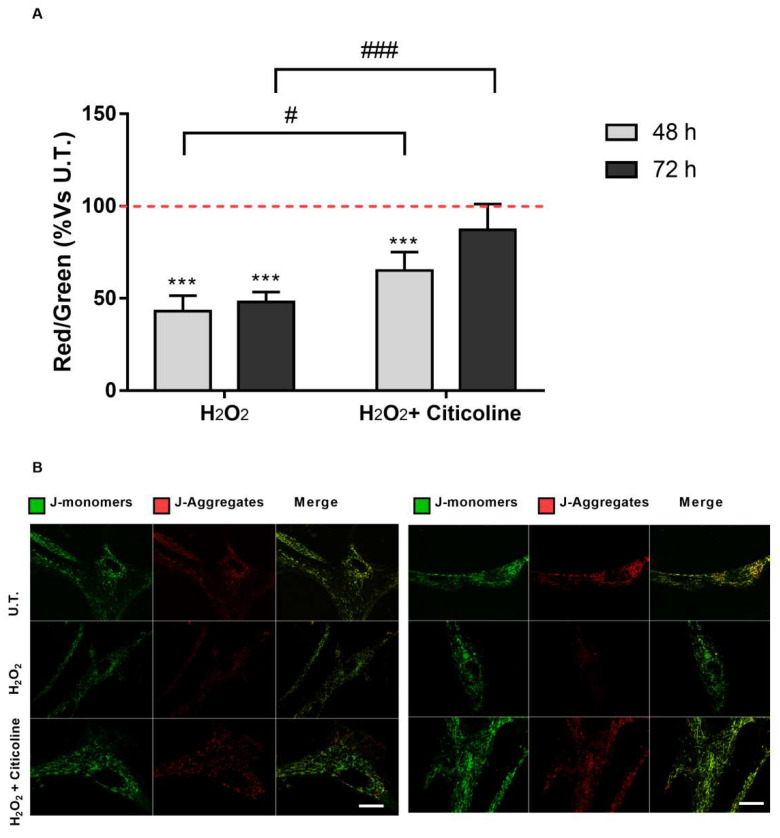
JC-1 assay. The mitochondrial transmembrane potential was monitored under all experimental conditions by JC-1 fluorochrome. (**A**) Quantitative analysis of the ratio of red/green fluorescent intensity was evaluated in HTMCs. The level of red and green intensity was calculated for at least 3 images from the same experimental conditions. Each measurement included ten areas of interest per image. (**B**) The figures depicted are representative of at least three confocal microscopy analyses of the mitochondrial membrane potential ± SD (scale bar marker corresponds to 20 µm). *** *p* < 0.001 vs. untreated (U.T.) HTMCs; ### *p* < 0.001, # *p* < 0.05 vs. treated HTMCs (two-way ANOVA followed by Bonferroni post-test).

Since the loss of mitochondrial transmembrane potential is the most significant event during apoptosis, the role of citicoline in the mitochondrial function was analyzed (Figure 4). The results showed that prolonged exposure of HTMC to H_2_O_2_ promoted mitochondria depolarization. However, citicoline significantly reduced the H_2_O_2_-induced progressive loss of the mitochondrial transmembrane potential (Figure 4A,B).

### 2.2. Citicoline Is Effective in Counteracting H_2_O_2_-Induced Cell Senescence and in Reducing the Release of Senescence-Related Pro-Inflammatory Cytokines

**Figure 5:**
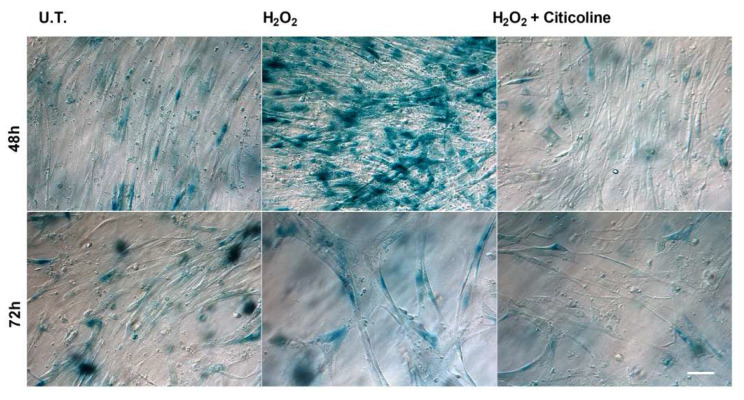
Senescence-associated β-galactosidase staining. HTMCs were stained for senescence-associated β-galactosidase after treatment with H_2_O_2_ alone or in combination with citicoline (scale bar marker corresponds to 40 µm). UT = untreated HTMCs.

Premature senescence is an acute and irreversible phenomenon induced by various stressors [37,38]. Thus, we analyzed the SA-β-gal activity, which is a well-accepted biochemical marker of cell senescence, in untreated and treated HTMCs. Representative cell images (Figure 5) showed that citicoline co-treatment did not affect either cell density or cell morphology in comparison to the untreated cells.

As shown in Figure 5, the 48 h treatment of HTMCs with 500 μM H_2_O_2_ induced a marked increase in SA-β-gal-positive cells that was strongly prevented by the combination with citicoline. Moreover, the 72 h treatment of HTMC with H_2_O_2_ induced a marked reduction in SA-β-gal-positive cells and a decrease in cell density. Also, in this case, citicoline supplementation was able to counteract the H_2_O_2_-mediated effects by decreasing SA-β-gal-labeled positive cells.

**Figure 6:**
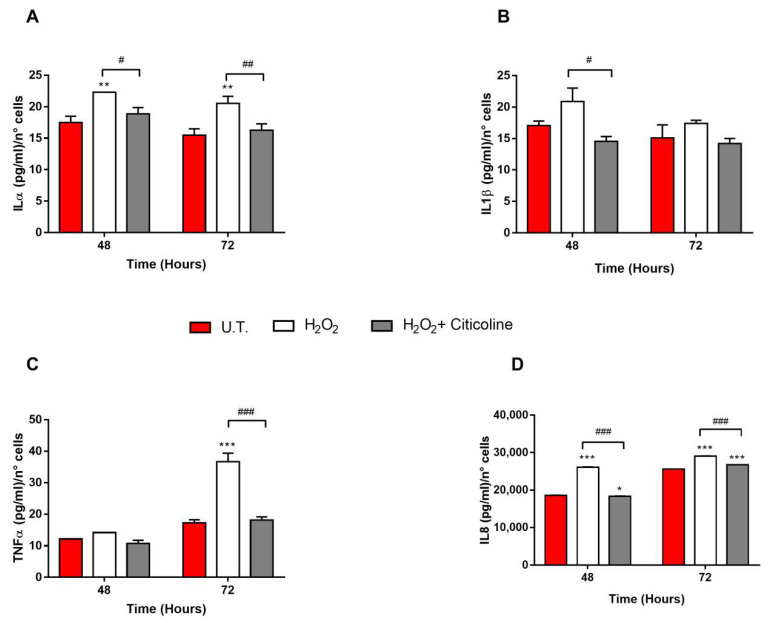
Measurement of pro-inflammatory cytokine release into HTMCs media. The levels of IL1α (**A**), IL1β (**B**), TNFα (**C**) and IL8 (**D**) released by HTMCs in culture media were analyzed by a Milliplex^®^ MAP kit (Merck KGaA). All collected samples were used directly, without dilution, and all samples, standards, and quality controls were assayed in accordance with the manufacturer’s instructions. The quantification of each cytokine level was extrapolated by referring the median fluorescent intensity (MFI) to a linear standard curve designed for each cytokine vs. pg/mL. Data, expressed as pg/mL and normalized with the number of live cells, represent the mean ± SEM of two separate experiments. *** *p* < 0.001, ** *p* < 0.01, * *p* < 0.05 vs. untreated (UT) HTMCs, ### *p* < 0.001, ## *p* <0.01; # *p* < 0.05 vs. treated HTMCs (two-way ANOVA followed by Bonferroni post-test).

The release of several inflammatory cytokines, including the major senescence-associated secretory phenotype (SASP) (i.e., IL1α, IL1β, TNFα and IL8) by untreated (U.T.) and treated HTMCs, was analyzed. Among them, only the levels of IL1α and IL8, in both timeframes tested, were found to be significantly higher in H_2_O_2_-treated HTMCs than U.T. and citicoline-cotreated ones (Figure 6A,D). Moreover, while a significant increase in H_2_O_2_-induced IL1β and TNFα secretion was also observed at 48 and 72 h, respectively (Figure 6B,C), the citicoline cotreatment showed its anti-inflammatory effects also in these cases showing a decreased, or even none, pro-inflammatory cytokine secretion.

## 3. Discussion

Glaucoma, AD, PD and Huntington’s disease all feature the same neurodegenerative pattern, i.e., mitochondrial dysfunction, oxidative stress, neuroinflammation, and lysosomal dysfunction [14]. From this perspective, to consider IOP management as the only therapeutic glaucoma treatment is not viable given that it has not shown itself to be sufficient in preventing retinal ganglion cell death in any patients [18]. The first pharmacological benefits of administered intramuscularly citicoline against glaucomatous optic nerve damage were described by Pecori Giraldi et al. [39] in 1989. To date, other ways of administering citicoline with encouraging results, i.e., oral and ocular routes, are available [10,16,17,40,41]. Recently, citicoline eye drops, namely, OMK1^®^, have shown their efficacy as a coadjuvant to hypotensive therapy in glaucomatous patients in terms of reducing the progression of the visual field and the loss of Retinal Nerve Fiber Layer [17]. Moreover, previous experimental and clinical studies on POAG patients report that therapy with citicoline eye drops reduces disease progression by acting as a neuroprotective/neuroenhancement drug [16,17,40,41]. However, since most of citicoline mechanism of actions, including the de novo synthesis of phospholipids and the increase in antioxidant defense, can be beneficial for other eye cells, and not only for RGCs, we hypothesized that it could exert a protective effect also for stressed TM. As is known, TM is the most sensitive tissue of the anterior chamber to the oxidative damage with reduced antioxidant defense [42]. A prolonged exposure to oxidative stress may result in senescence or/and injury to structural and functional TM components, including DNA, mitochondria, proteins, and membrane lipids [43]. Evidence shows that a glutathione-depleted TM along with a high concentration of hydrogen peroxide induces apoptosis as well as TM disruption and collapse [44,45]. Moreover, even though the progression of glaucoma-induced blindness is a complex event, the loss in phospholipid homeostasis negatively affects visual processing because phospholipids play a significant role in cell signaling and tissue physiology [46].

Therefore, a deeper understanding of the possible role of TM in POAG neurodegeneration can shed more light on the importance of using TM-targeted therapies.

To our knowledge, this is the first time that the cellular mechanisms underlying the cytoprotective action of citicoline eye drops have been investigated on HTMCs by using a customized in vitro model [35]. The herein-reported results indicate that, in the presence of H_2_O_2_-induced oxidative stress, citicoline eye drops considerably reduced the intracellular ROS amount and preserved viability of HTMC with respect to cells not treated with the compound (Figure 1A,B). Also, citicoline protected HTMCs from extrinsic and intrinsic apoptosis pathways induced via H_2_O_2_ (Figure 2A), probably by upregulating the anti-apoptotic proteins cIAP2 and XIAP (Figure 2B), and by restoring the mitochondrial transmembrane potential (Figure 4). Further evidence about the protective effect of citicoline against redox unbalance concerned both the lack of HSPs upregulation (Figure 2C) and prevention of premature senescence in cells challenged with H_2_O_2_ and stimulated with the compound (Figure 5). Remarkably, HSPs upregulation and premature senescence were clearly detectable in HTMCs challenged with H_2_O_2_ in the absence of citicoline.

Finally, citicoline modulated also the release of senescence-related pro-inflammatory cytokines (Figure 6) [37,38].

Although our in vitro platform is a rather simplified representation of any actual in vivo scenario and, overall, needs further improvement in the future, such an approach can provide important information on the TM-targeted activity of citicoline eye drops. The 3D culture model *per se* contributes to the so-called “closer-to-in Vivo” behavior of the cells compared to the conventional 2D one, whilst dynamic bioreactor systems allow for adding further complexity to cellular environments, encouraging both a cellular cross-talk through soluble agents and a constant nutrients supply [47,48,49]. Thus, we believe that our in vitro platform was able to highlight the potential efficacy of citicoline eye drops on stressed HTMCs. We speculate that the mechanism of action is likely through a phospholipid recovery given that phospholipid homeostasis plays a relevant role in guaranteeing the correct functioning of biological cell activity. Therefore, the observed protective action of citicoline, in addition to that already known and well documented for neurons and RGCs, could also be extended to other targets related to POAG, such as TM.

## 4. Materials and Methods

### 4.1. Cell Cultures

The HTMC primary cultures (number of batches: 3178, 3194), from donors aged between 74 and 79 years, and the Trabecular Meshwork Growth Medium (TMGM) both came from Cell APPLICATION INC. (San Diego, CA, USA). HTMCs, during the growth phase, were maintained with TMGM, whilst under experimental conditions. HTMCs were cultured with Dulbecco’s modified eagle’s medium (DMEM) containing a 1:1 mix of low and high glucose, 2 mM L-glutamine, antibiotics (0.5% gentamicin) and streptomycin (100 μg/mL), w/o fetal bovine serum [24,25,50], according to Keller et al. (2018) [51].

All cell cultures were found to be mycoplasma-free during regular checks with the Reagent Set Mycoplasma Euroclone (Euroclone).

A 3D-HTMC model was obtained by suspending 5 × 10^5^ cells in 200 μL Corning Matrigel Matrix (Corning Life Sciences, Tewksbury, MA, USA), and quickly seeded into the culture chamber of each bioreactor, LiveBox1 (LB1) (IVTech srl., Massarosa, LU, Italy). After polymerization at 37 °C, 1 mL of culture media was added and then replaced with fresh medium 24 h later [52].

After 24 h of seeding, the cells were first treated as described below and then were subjected to a bioreactor system, a sophisticated model of a milliscaled, multi-organ device in a single-flow configuration (LB1, IVTech srl) equipped with a peristaltic pump (LF, IVTech srl), to keep cells under dynamic conditions. The complete circuit diagram has been fully described elsewhere [25].

### 4.2. Citicoline Solution

Citicoline monosodium salt (Kyowa-Cho, Hofu City, Yamaguchi, Japan) was provided by Omikron Italia srl. (Italy). The citicoline solution was freshly prepared on the day of the experiment by dissolving 2 gr of citicoline monosodium salt in 100 mL of culture medium (2% *w*/*v*).

### 4.3. Experimental Conditions

The 3D-HTMCs were subjected to 500 µM H_2_O_2_ for 2 h per day under static conditions [25]. After incubation, the cell medium was removed and replaced with fresh medium or medium containing citicoline for an additional 2 h per day. Next, the untreated, H_2_O_2_-treated and H_2_O_2_ + citicoline-treated HTMCs were maintained under dynamic conditions for 20 h per day. These experimental treatments were prolonged up to 72 h.

### 4.4. DCF Assay

The antioxidant properties of citicoline were evaluated in terms of treated 3D HTMC ROS production, by dichlorofluorescein (DCF) assay.

3D HTMCs were exposed to non-fluorescent 2′,7′-dichlorodihydrofluorescein diacetate (H2DCFDA, Thermo Fisher Scientific Inc., Monza, Italy, Italy), which is able to permeate the plasma membrane and is reduced to the highly fluorescent 2′,7′-dichlorofluorescein [53]. The experiments were performed as described elsewhere [24] and each condition was analyzed 6 times. DCF emission was recorded on a fluorescent plate reader at excitation and emission wavelengths of 485 and 520 nm, respectively. The fluorescence intensity was extrapolated after subtracting the blank (Matrigel plus medium plus DCF) and the data were expressed as percentages of relative fluorescence units of treated vs. untreated HTMC cultures.

### 4.5. Viability Assessment

Alamar Blue assay (Thermo Fisher, Monza, Italy) was used for assessing the viability cells, according to the manufacturer’s protocol. Further method details are described elsewhere [35].

### 4.6. Apoptosis Array

The apoptosis pathway analysis was performed by the Human Apoptosis Antibody Array C1 (RayBio^®^; Norcross, GA, USA), according to the manufacturer’s protocol. The intensity of the protein array signals was analyzed using a BIORAD Geldoc 2000, and each protein spot was normalized against Positive Control Spots printed on each membrane.

The data analysis was conducted according to the protocol instructions of the Human Apoptosis Array C1, and the relative protein expression on different arrays was extrapolated by using the algorithm according to the Human Apoptosis Array C1 protocol [50].

### 4.7. Annexin V-FITC and PI Apoptosis Detection Kit

To evaluate cell death after 48 h and 72 h of experimental conditions, both the early and late apoptosis stages of 3D-HTMCs were analyzed using the Annexin V-FITC and PI apoptosis detection kit (Dojindo Molecular Technology, Inc., Rockville, MD, USA). Cells were stained with Annexin V-FITC and PI solution, as described elsewhere [35], and both apoptotic and necrotic cells were identified by confocal microscopy after 15 min of incubation in the dark at room temperature.

### 4.8. Mitochondrial Transmembrane Potential Analysis with the JC-1 Fluorochrome

The mitochondrial transmembrane potential of 3D-HTMC, treated as mentioned above, was analyzed by JC-1 fluorochrome, a marker of mitochondrial membrane potential, and then by an apoptosis trigger indicator, following the same steps described elsewhere [35]. Functional mitochondria exhibit a red J-aggregate signal compared to depolarized mitochondria, which show a green fluorescence due to the J-monomer signal. The red/green fluorescence signal ratio was analyzed after background subtraction. ImageJ-win 32 software was used for the intensity measurement of specific selected ROIs.

### 4.9. Senescence-Associated Beta-Galactosidase Activity Analysis

The cells were stained for Senescence-Associated beta-galactosidase (SA-β-gal) according to Dimri et al. [54]. Following experimental treatments, the 3D cultures were fixed using 0.5% (*v*/*v*) glutaraldehyde in PBS for 15 min at room temperature and, after two PBS washes, were incubated overnight at 37 °C with freshly prepared staining solution. The staining solution was composed of 1 mg ml^−1^ X-gal (Merck KGaA, Darmstadt, Germany) in 40 mM citric acid/sodium phosphate (pH 6.0) with 150 mM NaCl, 2 mM MgCl_2_, 5 mM potassium ferrocyanide and 5 mM potassium ferricyanide (all chemicals from Sigma Aldrich). After overnight incubation, the cells were again washed twice with PBS and imaged for the presence of the blue-colored staining using Leica DMI 3000 B.

### 4.10. Pro-Inflammatory Cytokines

To investigate the anti-inflammatory effects of citicoline, the cytokine profiling, released into the cell culture media of 3D-HTMCs after treatments, was analyzed by fluorescent bead-based technology, i.e., a Milliplex^®^ MAP kit (Merck KGaA). In brief, the supernatants were harvested at the end of each experimental procedure and stored at −20 °C until use. The levels of Interleukin (IL)-1α, IL-1β, IL-8, and Tumor Necrosis Factor alpha (TNFα) were analyzed in duplicate in 12 samples undergoing stimulation for 48 and 72 h. The method was performed according to the manufacturer’s instructions and the bead fluorescence readings were carried out by Luminex^®^ 200^TM^ (Merck KGaA).

### 4.11. Statistical Analysis

The data are presented as the mean ± SD or standard error. Statistical significance between the groups was assessed by one-way ANOVA or two-way ANOVA, followed by Bonferroni post-test. A *p*-value of *p* < 0.0001, *p* < 0.001, *p* < 0.01 and *p* < 0.05 was defined as indicating a statistically significant difference.

## Data Availability

Not applicable.
